# Effect of Pruning Young Branches on Fruit and Seed Set in Cassava

**DOI:** 10.3389/fpls.2020.01107

**Published:** 2020-07-23

**Authors:** Marcela Pineda, Benchi Yu, Yinong Tian, Nelson Morante, Sandra Salazar, Peter T. Hyde, Tim L. Setter, Hernán Ceballos

**Affiliations:** ^1^ CGIAR Research Program on Roots Tubers and Bananas (RTB), The Alliance of Bioversity International and the International Center for Tropical Agriculture (CIAT), Cali, Colombia; ^2^ Cassava Program, Guangxi Subtropical Crops Research Institute, Nanning, China; ^3^ Section of Soil and Crop Sciences, School of Integrative Plant Science, Cornell University, Ithaca, NY, United States

**Keywords:** apical dominance, feminization, hermaphrodite flowers, inbreeding, short breeding cycle, speed breeding

## Abstract

Flowering in cassava is closely linked with branching. Early-flowering genotypes branch low and abundantly. Although farmers prefer late flowering genotypes because of their erect plant architecture, their usefulness as progenitors in breeding is limited by their low seed production. In general, the first inflorescence aborts in cassava. Preventing this abortion would result in early production of seeds and make cassava breeding more efficient. The objective of this study was to assess if pruning young branches prevents the abortion of first inflorescences and promotes early fruit and seed set. Four genotypes with early, late, very late, and no flowering habits were grown under an extended photoperiod (EP) or normal dark night conditions (DN). Additional treatments included pruning young branches at the first or second flowering event and spraying (or not) benzyladenine (BA) after pruning. One genotype failed to flower and was not considered further. For the remaining genotypes, EP proved crucial to induce an earlier flowering, which is a pre-requisite for pruning. Total production of seeds in EP plots was 2,971 *versus* 150 in DN plots. For plants grown under EP, the average number of seeds per plant without pruning was 3.88, whereas those pruned produced 17.60 seeds per plant. Pruning at the first branching event led to higher number of seeds per plant (26.25) than pruning at the second flowering event (8.95). In general, applying BA was beneficial (38.52 and 13.98 seeds/plant with or without spraying it, respectively). The best combination of treatments was different for each genotype. Pruning young branches and applying BA in the first flowering event not only prevented the abortion of inflorescences but also induced the feminization of male flowers into hermaphrodite or female-only flowers. The procedures suggested from this study (combining EP, pruning young branches, and spraying BA), allowed the production of a high number of seeds from erect cassava genotypes in a short period. The implementation of these procedures will improve the breeding efficiency in cassava.

## Introduction

Cassava (*Manihot esculenta* Crantz) has significant economic relevance, particularly in the lowland tropics. Its main product is the starchy roots that are harvested, usually, about 12 months after planting (**MAP**). It is a fundamental food security crop in many regions of the world, particularly Sub-Saharan Africa. It is also an important industrial crop, the second source of starch worldwide (Stapleton, 2012).

Cassava is a perennial species usually grown as an annual crop. What is commonly identified as a male flower is actually an inflorescence of 10 single-stamen flowers. These inflorescences, known as cyathia, are protected by petal-like bracts. Male and female cyathia will be treated in this article as if they were single flowers as the distinction is only relevant from the botanical point of view. Cassava is a diclinous and monoecious species producing either female (pistillate) or male (staminate) flowers in terminal inflorescences (racemes or panicles) within the same plant. Occasionally, hermaphrodite flowers can be observed but at very low frequency (Perera et al., 2012).

Inflorescences always develop at the apex of the stem. Sprouting of the buds below the inflorescence allows further growth of the plant. To the naked eye, flowering and branching appear to take place simultaneously (Gonçalves Fukuda et al., 2002). Male flowers are considerably more numerous and develop in the upper section of the inflorescence (Kawano, 1980; Ogburia and Okele, 2001; Gonçalves Fukuda et al., 2002; Perera et al., 2012; Ramos Abril et al., 2019). Female flowers are found in the proximal branches of the inflorescence and their anthesis occurs about 14 days earlier than that of male flowers (protogyny).

Sexual reproduction, the key requirement for crop breeding, is common and relatively easy to achieve in cassava (Kawano, 1980; Alves, 2002). Some genotypes flower early and several times, starting two or three MAP, and up to six times during a growing cycle. These genotypes develop a bushy type plant architecture. Other genotypes flower little and late (or not at all), resulting in an erect plant architecture. Although time and frequency of flowering are clearly under genetic control, there is also strong environmental influence. For example, early studies suggested that flowering in cassava was favored by longer photoperiods and cooler temperatures (de Bruijn, 1977; Keating, 1982).

Early branching genotypes tend to produce progenies that flower early and branch low. This type of plant architecture facilitates greatly the breeding work because botanical seed is produced early and in abundance. However, farmers generally prefer erect varieties and breeders increasingly favor late and scarcely flowering progenitors. The production of botanical seed from erect genotypes is sparse, slow, and expensive. Perhaps one of the most important areas of research to accelerate genetic gains in cassava, therefore, would be the development of technologies that accelerate and facilitate the production of seed from erect cassava genotypes in crossing nurseries.

Considering the vegetative reproduction of cassava, efficient sexual reproduction is not essential for this species. This has proven to be the case (Jennings, 1963; Ogburia and Okele, 2001; Ramos Abril et al., 2019). In most systems the shoot tips control the development of branches through a complex process of auxins and cytokinin synthesis and transport, interacting with environmental and epigenetic factors (Dewitte et al., 1999; Dun et al., 2006; Prusinkiewicz et al., 2009; Costes et al., 2014). Two important changes take place when cassava transitions to flowering: the apical meristem switches to produce a terminal inflorescence and the growth of lateral vegetative branches is no longer inhibited. The branches that emerge in the first flowering event quickly outgrow the inflorescence, which seems incapable of exerting apical dominance and usually aborts. Therefore, the earliest botanical seed that can be produced in crossing nurseries comes from the second flowering event. This feature is relevant for cassava breeding because the second flowering event in erect genotypes happens, at best, late during the first year of growth. Late flowering also implies a logistic challenge because flowers are often found more than two meters above ground. In practical terms, breeders need to wait two years to get seed from a planned cross involving an erect genotype.

There have been important progress manipulating flowering biology of cassava in the past few years. Earlier flowering was achieved through grafting (Ceballos et al., 2017; Silva Souza et al., 2018) and the extension of the photoperiod (Pineda et al., 2018). Recently, the anti-ethylene growth regulator silver thiosulfate was reported to reduce abortion of early inflorescences, and to promote flower development and longevity (Hyde et al., 2019). Finally, the combination of extended photoperiod with plant growth regulators (benzyladenine and/or silver thiosulfate) has been tested, but with negative results (CIAT, unpublished data).

The limitations for the production of botanical seed from crosses between erect genotypes are further increased in subtropical regions of the world due to the short growing cycle (compared with low latitude environments). An unpublished innovative approach was developed at Guangxi Subtropical Crops Research Institute (GSCRI) (Nanning, China). The key feature of this approach is the removal of lateral branches as soon as the transition to flowering has taken place. Pruning young branches strengthens the apical dominance of the terminal inflorescence and prevents its abortion. Moreover, the first inflorescence seems to achieve unprecedented development with a large number of fertile flowers.

The objectives of the present study were: ***i)*** to validate the effect of pruning young branches in tropical environments using South American cassava germplasm under normal and extended photoperiod conditions; ***ii)*** to compare the effect of pruning lateral branches at the first and second flowering events; ***iii)*** to assess the effect of plant growth regulator, benzyladenine, in combination with pruning; and ***iv)*** to monitor any observable change, in addition to fruit and seed production, caused by pruning and/or plant growth regulators.

## Materials and Methods

### Location

All data were collected at CIAT’s Experimental Station, in Palmira, Valle del Cauca, Colombia. This site is located less than four degrees north of the Equator. The duration of the photoperiod is therefore uniform throughout the year. In some cases, plants were grown under extended photoperiod conditions (EP) which began as soon as the stem cuttings were planted. The altitude of this location is 965 meters above sea level. Night temperatures tend to be lower than at locations in the same latitude but at sea level. Average maximum and minimum temperatures through the year are 30.1 ± 2.7°C and 19.2 ± 1.2°C, respectively.

### Germplasm

Four genotypes were chosen for this study based on their flowering patterns. Three of these genotypes showed contrasting response to grafting and/or extension of photoperiod (Pineda et al., 2018): **GM 971-2** begins flowering abundantly 2-3 MAP with a marginal response to EP; **CM 4919-1** is a late flowering genotype. Under normal, dark-night conditions (DN) it flowers for the first time about nine MAP, but under EP it begins flowering 5 months earlier. **SM 3348-29** branches and flowers only after grafting on an early branching understock (Ceballos et al., 2017) when grown under DN. It also responds to EP, but flowering is always later than in CM 4919-1. **GM 3893-65**, or “*Asparagus*” cassava, has leaves without petiole and does not flower within a normal growing cycle. It responds marginally to EP. These genotypes, therefore, provided a wide range of flowering patterns.

### Field Management

Field management followed the standard procedures for cassava at CIAT. A mixture of the pre-emergence herbicides Karmex (Diuron Adama, Colombia) and Dual Gold (S-metolachlor, Syngenta, Colombia) was applied 4–7 days before planting. Manual weeding was made as necessary. Plots were uniformly fertilized following standard procedures (Cadavid-L, 2012). Irrigation was provided *via* surface/gravity distribution also as required. Pests pressure, particularly whiteflies (*Aleurotrachelus socialis*), was monitored constantly and maintained under control.

### Experiment 1

**Figure 1:**
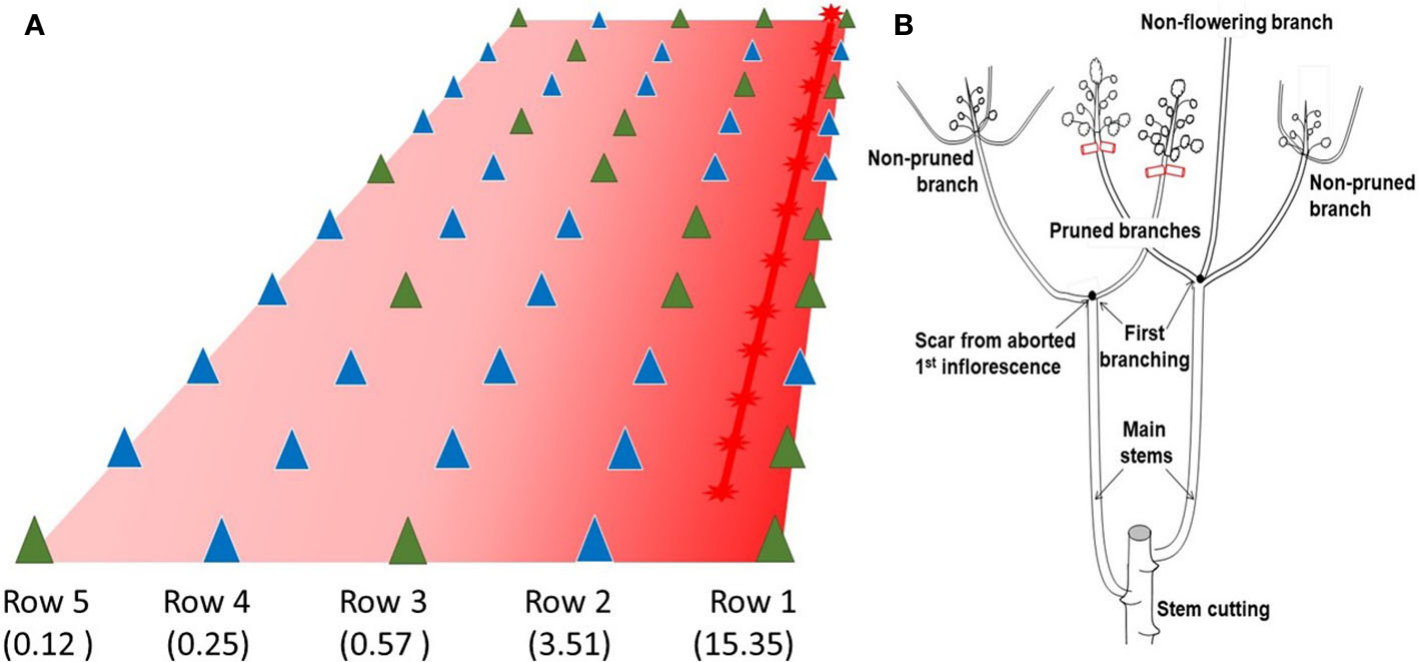
Illustration of the planting design of Experiment 1. **(A)** Red light was provided by a LED tape positioned on top of row 1. The gradient in color intensity indicates that plants on the right were exposed to a higher flux density than those on the left (within parenthesis average lx on each row). Triangles indicate the position of a plant. Blue triangles indicate pruned plants. Green triangles indicate non-pruned plants. **(B)** Description of the pruning protocol. In CM 4919-1 sprouting of the stem cutting typically produces two main stems. After the 1^st^ flowering event, the plant produces two to three branches. The apical shoots in these branches were randomly selected for pruning when the 2^nd^ flowering took place. Some of the branches were left untouched, while others were pruned for pair-wise comparison. Occasionally, some branches did not flower.

The early and innovative experiences at GSCRI in Nanning suggested that, under prevailing subtropical conditions and using germplasm developed in SE Asia, pruning young branches prevented the abortion of inflorescences and enhanced flower production, particularly from the first branching event. However, for pruning to be effective, flowering needs to have been already triggered. This requirement is a serious limitation for erect plant genotypes. This experiment was carried out validate the effect of pruning on plants which had been exposed to EP. Five rows with 10 plants each were planted with clone CM 4919-1, characterized by a strong response to EP (Pineda et al., 2018). Row spacing was 1.2 m and plant to plant distance within the row was 0.5 m. Red light was provided by a 5 m long red LED tape (wavelength peak around 620–640 nm) placed on top of the first row (Row 1 at the right of **Figure 1A**). This arrangement provided a gradient on light intensity which was maximum at the row immediately below the LED tape and minimum at the row farther away. Differences in light intensity across rows spread the peak of flowering events which facilitated the pruning logistics. However, every plant received the minimum light intensity required to elicit flowering in cassava (estimated to be around 0.02 μmol/m^2^/sec). Illumination began soon after planting and, as plants grew, the LED tape was raised. Lights were turned on at sunset and turned off at sunrise.

**Figure 2:**
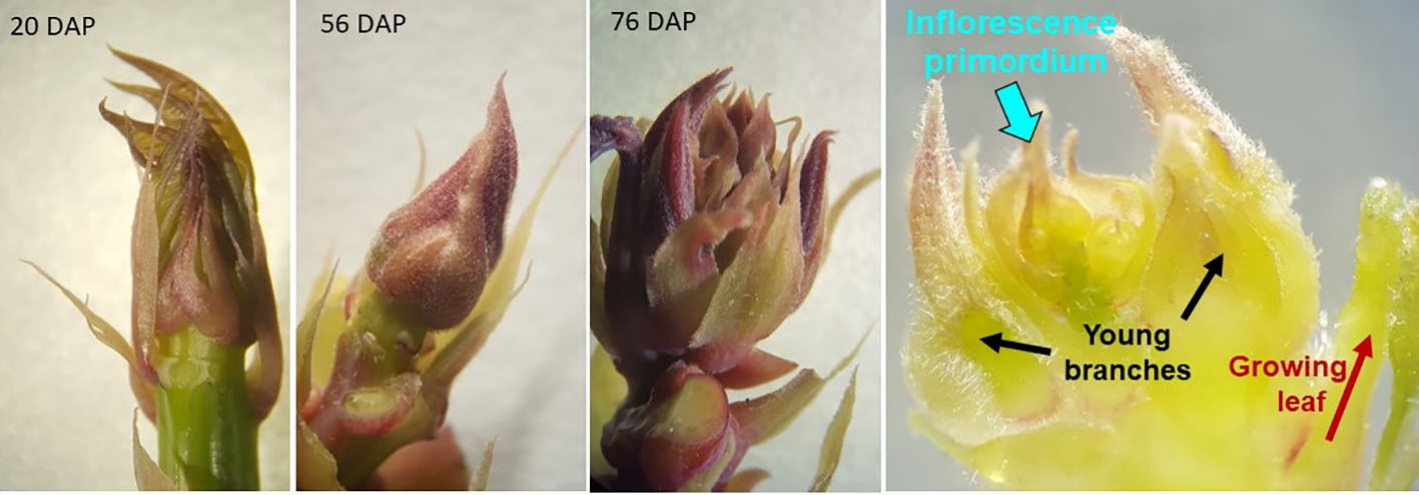
Illustration of the apical meristem of GM 971-2 as it shifts from vegetative to reproductive mode. The growing tip has a tear-shaped appearance through 56 days after planting (DAP), but assumed a globular shape by 76 DAP. For the photograph on the right, taken with a stereoscope, some young leaves have been removed to expose the inflorescence primordium.

Experiment 1 was, in many ways, an exploratory experience. Early identification of apical shoots that had undergone the transition to flowering is difficult for an unexperienced person. **Figure 2** illustrates the distinctive change in the shape of the shoot when flowering has been induced. Shoots during vegetative growth have a tear shape, which turns globular after the initiation of flowering. The unpublished experiences at GSCRI indicated that branches must be removed early, when their size is small (5–8 mm). It was necessary, therefore, to gain experience in pruning the branches without damaging the inflorescence, which is not a trivial task. A video document (https://youtu.be/hHHvaCzvB0E) illustrating the pruning procedure is available. Pruning was carried out only at the second flowering event to maximize the number of growing shoots per plant (**Figure 1B**). A pair-wise design was used to analyze data (Steel and Torrie, 1960). Comparisons were made only between pruned and non-pruned branches in the same plant.

### Experiment 2

Four experimental genotypes were planted (GM 971-2, CM 4919-1, GM 3893-65, and SM 3348-29**)** on June 6, 2018. Each treatment x genotype combination was planted in a single row plot with 10 plants. Rows were spaced 1.5 m and plant to plant distance within the row was 0.5 m. Basically, the same pruning experiment was duplicated. In one case plants grew under DN and in the other with EP, which began the day of planting. Illumination of plants lasted all night long. There were five treatments implemented in each of the four genotypes (nested within each of these two photoperiod conditions): ***i)*** No pruning; ***ii)*** Pruning in the first branching event; ***iii)*** Pruning in the first branching event, combined with the application of benzyladenine (BA); ***iv)*** Pruning in the second branching event; and ***v)*** Pruning in the second branching event, combined with the application of BA. The four genotypes serve as replications for the five pruning treatments. Rather than controlling environmental variation (as blocks do in a randomized complete block design) the replications in the present study address the issue of genetic variation, which in the case of flowering, is a critical factor to consider.

The source of light used for the EP treatment was 50W LEDs with reflectors fixed at 3 m above ground in a square grid 4.5 m apart. Results from previous evaluations (unpublished data) indicated that every plant received a stimulus in excess of the 0.02 μmol/m^2^/sec required to elicit earlier flowering. The maximum light intensity at ground level immediately below the 50W LED was 0.10 μmol/m2/sec. The illumination from neighboring reflectors overlapped slightly. Light intensity was not uniform in the experimental area and with respect to time because, as the plants grew and became closer to the lamps, light intensity at the apex of the plants increased. Light intensity and quality (wavelength peak around 620–640 nm) had negligible effect on photosynthesis and temperature at the apex of the plants. A 50 ppm (w/v) BA solution was prepared mixing commercial MaxCel (Valent BioSciences, Libertyville, Illinois, USA) with distilled water. The solution was sprayed (5 ml of the solution) in the top of plants that required the BA treatment immediately after pruning the lateral vegetative branches. BA was applied weekly after the day of pruning until the transition from flowers to fruits was observed. BA was not applied to unpruned plants. Data was taken individually on each plant. This trial was kept for 300 days in the field. Flowers were not covered and were left untouched for open pollination to take place.

### Data Recorded

Plots were visited daily and records were taken when a plant began flowering and branched. The number of days from planting to first branch was registered individually on each plant, as well as height of first branching. Number of nodes to first branching and number of fruits and seeds were also registered individually on each plant.

### Statistical Analysis in Experiment 2

**Figure 3:**
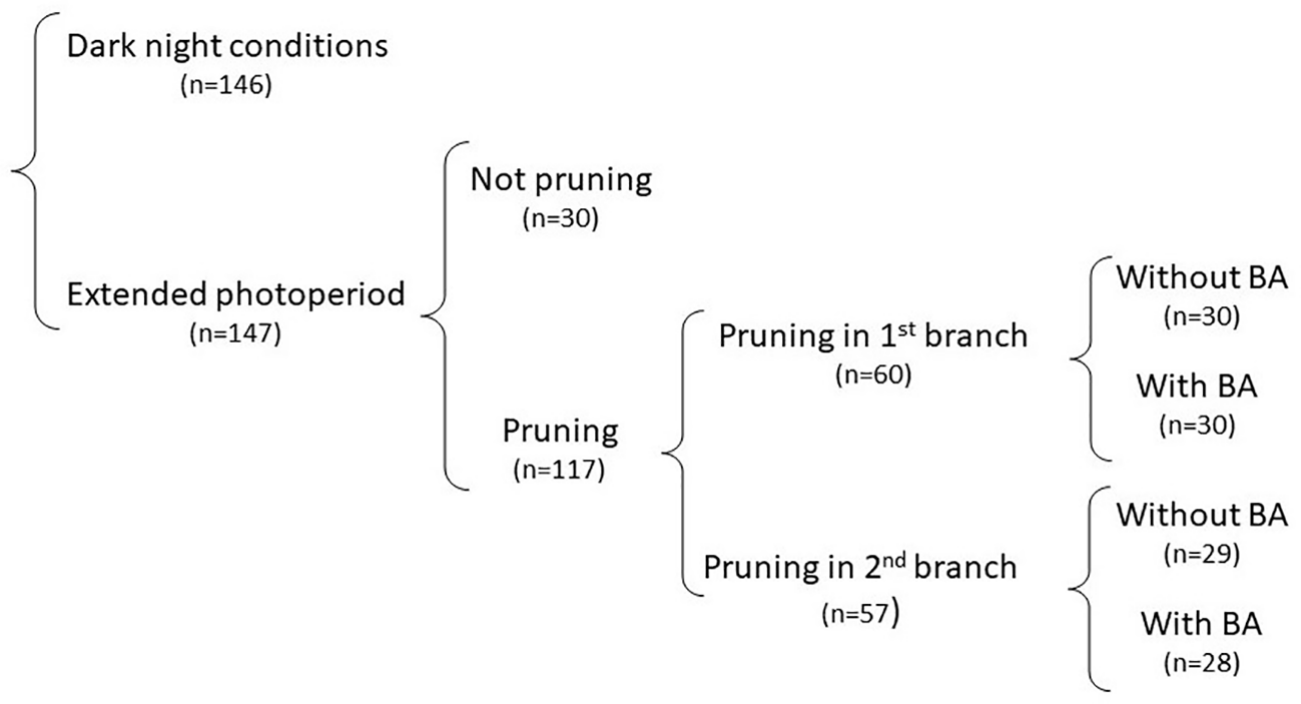
Description of the way data in Experiment 2 were analyzed. The impact of pruning and BA application under normal photoperiod could not be properly assessed because of limited occurrence of flowering. The analysis, therefore, focused on data from treatments under extended photoperiod. The hierarchical classification of treatments allowed for successive orthogonal contrasts following a nesting approach, which are described in this figure. Sample sizes for each contrast have been included.

Data were taken on individual plants. The experimental units, therefore, were the individual plants within a 10-plant plot. Each pruning treatment x clone combination was planted in a single row and randomly allocated within each of the two photoperiod conditions used in the study. Plots were visited daily and records were taken when a plant began flowering and branched. Statistical analyses were conducted with SAS (SAS, 2008). Analysis of variance was made through PROC GLM. Successive orthogonal contrasts in the ANOVA followed a nesting approach within a hierarchical classification (Steel and Torrie, 1960. **Figure 3**). Biological features in the study, in a way, imposed the nesting approach. EP was included to elicit flowering, which is a requirement for pruning. BA was applied only after pruning to target the developing inflorescences and flowers. The first contrast was between the averages of the two photoperiod conditions. The entire data set was then divided into two subsets for the DN and EP conditions and further analyses were conducted separately. The second contrast, pruning *versus* not pruning, was nested within each photoperiod condition. Similarly, the third contrast (pruning in the first or second branching event) was nested within the pruning-EP or pruning-DN combination. Finally, the fourth and fifth contrasts (spraying BA or not) were nested within pruning in the first or second branching events, respectively. All sources of variation were considered fixed (except the plant-to-plant variation within a plot). Type III sum of squares were used for the ANOVA. LSD test was used to assess differences among averages.

## Results

In general, the different trials grew well without relevant problems. Environmental conditions were representative for the location used. There was no major biotic problem, except for an unusual mite pressure that was quickly controlled.

### Experiment 1

Fifty stem cuttings were planted and 49 sprouted producing a total of 95 main stems (3 plants produced only one while the remaining 46 plants produced two main stems). On average each main stem produced 2.25 branches in the first flowering event, resulting in a total of 216 branches. It was the apex of some of these branches that were pruned during the second flowering event (**Figure 1B**). A total of 191 branches flowered (173 of them between December 18, 2017 and January 18, 2018) and 121 of them were left untouched, while the remaining 70 were pruned. As expected, flowering in rows closer to the source of light was slightly earlier than those farther. Average number of days to the second flowering events were 152.1; 149.4; 154.3; 160.7; and 158.6 respectively for rows 1 through 5, corresponding to progressively more distant from the source of light. Since flowering was induced in every row, it is clear that light intensity even in row 5 was above the stimulus threshold (CM 4919-1 under DN does not flower at all at this age). The number of pruned shoots were 8, 17, 13, 16, and 16, respectively for rows 1 to 5. The decision to prune or not was random and, to great extent, determined by how early young shoots that had already flowered could be timely identified. On average the number of non-pruned and pruned branches in the 30 plants used for the pair-wise contrast were 2.6 and 2.1, respectively. **Table 1** summarizes the result of this exploratory experiment. The average number of fruits and seeds (1.19 and 2.72, respectively) in pruned shoots was significantly larger than in those that were not (0.75 and 1.53).

**Table 1:** Pair-wise comparisons between pruned and non-pruned branches from the same plant in Experiment 1.

Response variables	Average (Number)			t-Test	P-value
	Pruned	Not pruned	Difference		
Number of fruits	1.19	0.75	0.44	2.31	0.03
Number of seeds	2.72	1.53	1.18	2.83	0.01

Results are based on 30 plants where both treatments were available.

The results obtained from Experiment 1 were encouraging. Pruning in the second flowering event of CM 4919-1, grown in tropical environments under EP produced significantly higher number of seeds. More importantly, it allowed personnel to gain experience in the process of identifying shoots that had undergone the transition to flowering and in the pruning procedure itself.

### Experiment 2

**Figure 4:**
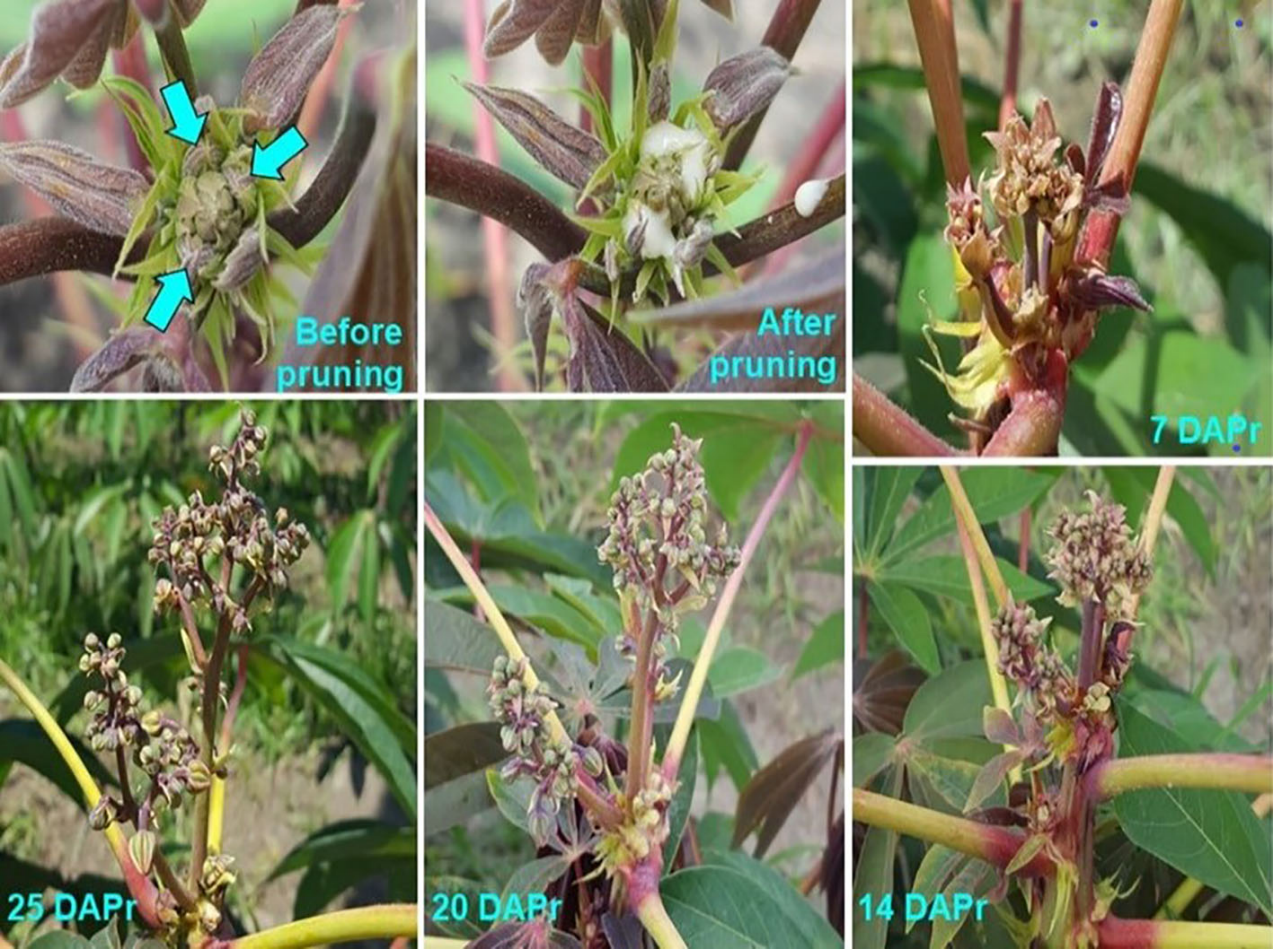
Effect of pruning a cassava plant. The photographs on the top left shows a growing tip before and after pruning (GM 971-2). The three young branches have been highlighted with arrows. Latex exudates from the scars after branch removal. The remaining photographs show the growth of an inflorescence from CM 4919-1 that, without pruning, would have aborted. By 25 days after pruning (DAPr) one female flower is already approaching anthesis.

Data from 390 plants were obtained (from the 400 stem cuttings planted). The experience gained during Experiment 1 was critical for a timely identification of apical shoots that had undergone the transition to flowering and the successful pruning procedure. **Figure 4** illustrates the key stages in the process. Plants from the “asparagus” cassava (GM 3893-65) failed to flower even under EP and thus could not be pruned. Information from this clone was not useful and was not considered in the contrasts presented in **Table 2**. This finding was consistent with genotypic responses to extended photoperiod which were previously reported (Pineda et al., 2018).

**Table 2:** Relevant averages from Experiment 2.

Description	Height of 1^st^	Days to 1^st^	Nodes to 1^st^	Fruits per	Seeds per
	branch (cm)	branch (#)	branch (#)	plant (#)	plant (#)
**Effect of extending the photoperiod**					
Significance of F-test	<0.0001	<0.0001	<0.0001	<0.0001	<0.0001
Normal photoperiod	201.5a	243.2a	130.8a	0.5b	1.0b
Extended photoperiod	87.1b	103.8b	48.1b	8.4a	20.0a
**Effect of pruning (under EP)**					
Significance of F-test	0.738	0.696	0.972	0.0094	0.0148
Not pruned	84.6a	105.5a	48.2a	2.1b	5.2b
Pruned	87.8a	103.3a	48.1a	9.9a	23.8a
**Contrast between pruning in the 1^st^ and 2^nd^ branching (under EP)**					
Significance of F-test	0.582	0.917	0.978	<0.0001	0.0002
Pruned at ^1st^ branch	91.9a	103.0a	47.7a	14.7a	35.0a
Pruned at 2^nd^ branch	83.4a	103.7a	48.4a	5.0b	12.0b
**Effect of applying BA (in plants pruned in the 1^st^ flowering event, under EP)**					
Significance of F-test	0.938	0.617	0.697	0.007	0.012
No BA applied	91.4a	104.8a	48.7a	7.8b	18.6b
BA applied	92.4a	101.2a	46.7a	21.5a	51.4a
**Effect of applying BA (in plants pruned in the 2^nd^ flowering event, under EP)**					
Significance of F-test	0.957	0.843	0.866	0.014	0.015
No BA applied	83.7a	103.0a	48.8a	2.5b	5.7b
BA applied	83.1a	104.4a	48.0a	7.5a	18.6a

Analysis of data followed a nested classification approach. Averages followed by different letters are statistically different based on LSD test at P < 0.05. The probability of the F-tests for the orthogonal contrasts is also presented. Contrasts within DN have been omitted because very few plants flowered and pruning was thus only possible occasionally.

The ANOVA (table not presented) indicated that the contrasts between DN and EP were highly significant (P < 0.0001) for every response variable: height of 1^st^ branch (HEIGHT); days to first flowering/branching (DAYS), number of nodes to first branching (NODES); number of fruits per plant (FRUITS), and number of seeds per plant (SEEDS). Only 32% of the plants growing under DN eventually flowered. Since flowering is a pre-requisite for pruning, data for the different pruning alternatives within DN sample sizes were unbalanced and limited. No further analysis of these data from DN is presented. On the other hand, results under EP allowed a useful assessment on the effect of pruning and application of BA on fruit and seed production. The analysis of data was based on a successive nesting of each source of variation on the others. This approach allowed relevant pairwise orthogonal contrasts between the different treatments, which are illustrated, along with the respective sample sizes, in **Figure 3**.

The first averages being compared (top of **Table 2**) are those across the five pruning treatments, three genotypes, and 10 plants per row (≈150 data points each, **Figure 3**). These results demonstrate the impact of extending the photoperiod to induce earlier flowering in different genotypes (except for GM 3893-65). Under EP, plants flowered lower on the stem (87.1 *vs* 201.5 cm) and earlier (103.8 *vs* 243.2 days to first flowering) than plants growing under DN. FRUITS and SEEDS per plant were significantly higher in EP compared with DN (**Table 2**). However, assessing the impact of EP was not the main objective of this work. EP was used to induce flowering which is a requirement for pruning.

As expected, HEIGHT, DAYS, and NODES were not different in any of the contrasts presented. This makes sense because every treatment in the study (except duration of photoperiod) was initiated after the first branching had occurred.

The most relevant information in **Table 2** is presented in the right two columns (FRUITS and SEEDS). As already stated, EP resulted in significantly higher total production of seeds compared with DN (2,971 *versus* 150, data not shown). Since these differences are across the five pruning treatments, they properly reflect the impact of extended photoperiod. The impact of pruning can be easily visualized looking at the data within EP.

**Figure 5:**
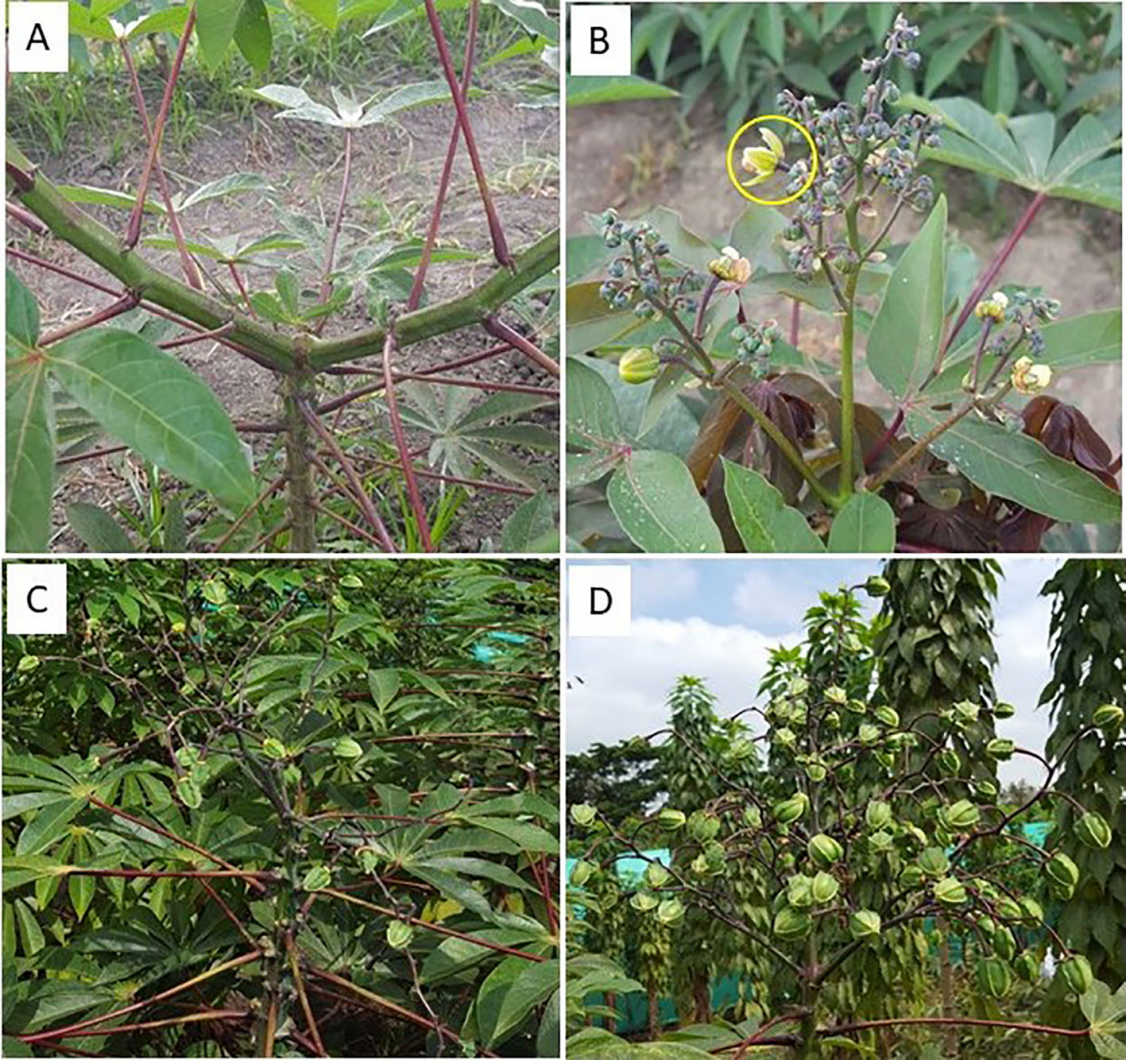
Effect of pruning in two cassava genotypes. **(A**, **B)** Photographs were taken from GM 971-2: without pruning **(A)** and after pruning in the first branching event **(B)**. A scar has been left as a result of the typical abortion of the first flowering event without pruning **(A)**. In contrast, the inflorescence is developing well and several flowers have already undergone anthesis after pruning **(B)**. **(C, D)** show plants from SM 3348-29 pruned in the first branching event: without **(C)** or with **(D)** application of BA.

Under EP, FRUITS and SEEDS from non-pruned plants were respectively 2.1 and 5.2, whereas those from pruned plants were 9.9 and 23.8, respectively. These results indicate that, by the time the experiment had been completed 300 days after planting, the best results were obtained in plants growing under EP that have been pruned. The effects of pruning can be visually appreciated in the photographs presented in **Figure 5**.

The following contrast presented in **Table 2** is for plants pruned in the first *versus* the second branching event. FRUITS values were 14.7 and 5.0 for plants pruned, respectively, in the first or second branching event. Similarly, SEEDS averages were 35.0 and 12.0 from plants pruned in the first and second branching, respectively. It is clear, therefore, that across genotypes, pruning in the first branching event results in greater seed production.

The final contrasts in **Table 2** assess the effect of BA application nested within pruning in the first or second branching event. For the former, the impact is striking with averages of 7.8 and 21.5 FRUITS without and after BA application, respectively. Similarly, averages for SEEDS were 18.6 and 51.4, without or with BA, respectively. **Figures 4C, D** illustrate the impact of BA spraying on fruit development in SM 3348-29. FRUITS and SEEDS values in plants pruned in the second flowering event were also significantly higher when BA was applied (bottom of **Table 2**). However, the benefits of BA application in plants pruned at the second branching were not as large as those in plants pruned earlier. There are two additional observations worth noting from **Figure 5B**: ***i)*** there is a female flower on top of the inflorescence (yellow circle) that would typically be male based on its position; and ***ii)*** the orderly evolution of flowers undergoing anthesis from bottom to top has been clearly disrupted.

**Figure 6:**
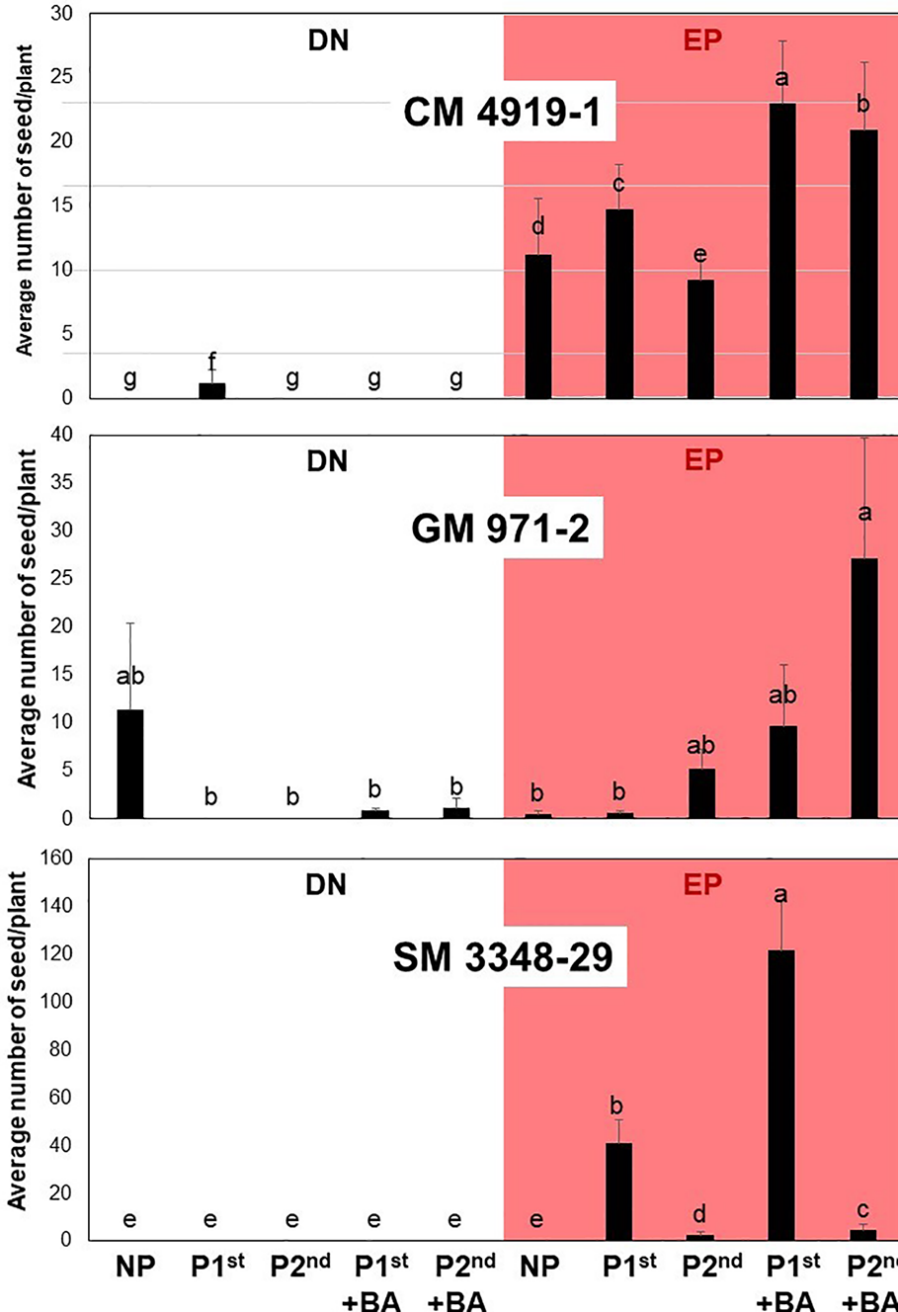
Average number of seed per plant produced under different treatments in three cassava genotypes. For each plot, the white and reddish area presents results from plants growing under dark-night (DN) and extended photoperiod (EP) conditions, respectively. NP (not pruned); P1^st^ (pruned in the first flowering event); P2^nd^ (pruned in the second flowering event); +BA (BA sprayed after pruning). The ten treatments listed at the bottom are the same for the three plots.

Results presented in **Table 2**, for clarity’s sake, are averages across genotypes. Individual responses (average seed per plant) to different growing conditions from each genotype (except for GM 3893-65) are summarized in **Figure 6**. Within each photoperiod condition, five different treatments were evaluated (from left to right): no pruning, pruning in the first or second branch (without BA), and pruning in the first branch or second branch (with BA).

The simplest results to describe are those from SM 3348-29. No plant from this genotype produced seeds under DN. Non-pruned plants from SM 3348-29 growing under EP failed to produce seed as well. In contrast, the largest number of seed per plant obtained across the entire experiment came from plants of this clone, pruned in the first branching event, and with the addition of BA (almost 125 seeds per plant). This is, indeed, a remarkable result. Even for early and profuse flowering genotypes (see results from GM 971-2), the production of this number of seeds would be difficult. Pruning plants of SM 3348-28 in the first flowering, without BA spraying, offered the second-best result, with more than 30 seeds per plant. These two averages were significantly different (P < 0.05). All the other treatments resulted in significantly lower averages. The unquestionable conclusion is that the combination of EP, pruning in the first branching event with the additional spraying of BA maximizes the early production of seed of this very late flowering cassava genotype.

The top of **Figure 6** presents results from CM 4919-1, which are also relatively easy to describe. This genotype, as was the case for SM 3348-29, responded well to EP. There was basically no production of seed under DN (except for plants pruned in the first branching event without BA spraying). The best two treatments for CM 4919-1 resulted in more than 20 seeds/plant and were obtained by pruning at first or second branching, with BA spraying. Pruning at the first branching event without BA or not pruning at all provided a second tier of averages (between 10 and 15 seeds/plant). The statistical significance of these differences is detailed in **Figure 6**.

Results from GM 971-2 contrast from those of the previous genotypes. This is the only clone among the germplasm evaluated that flowered often under DN (**Figure 6**). Pruning plants under DN was not beneficial. Under EP, pruning in the second branching event (with BA spraying) resulted in significantly higher averages compared with all the other treatments from GM 971-2. Since the first branching event in this clone takes place relatively early, there would be only a few leaves below it. This reduced number of leaves would not be enough to sustain abundant fruit set in plants pruned in the first flowering event. The averages presented in **Figure 6** come from the 10 plants representing each clone x treatment combination. These 10 plants were all planted together in a single row. It is acknowledged that there was no replication for the clone x treatment combinations. The environmental variation affecting these averages, however, is considered to be negligible because of the relatively small area (<130 m^2^ for EP plots) and the uniformity of soils in the experimental station.

**Figure 7:**
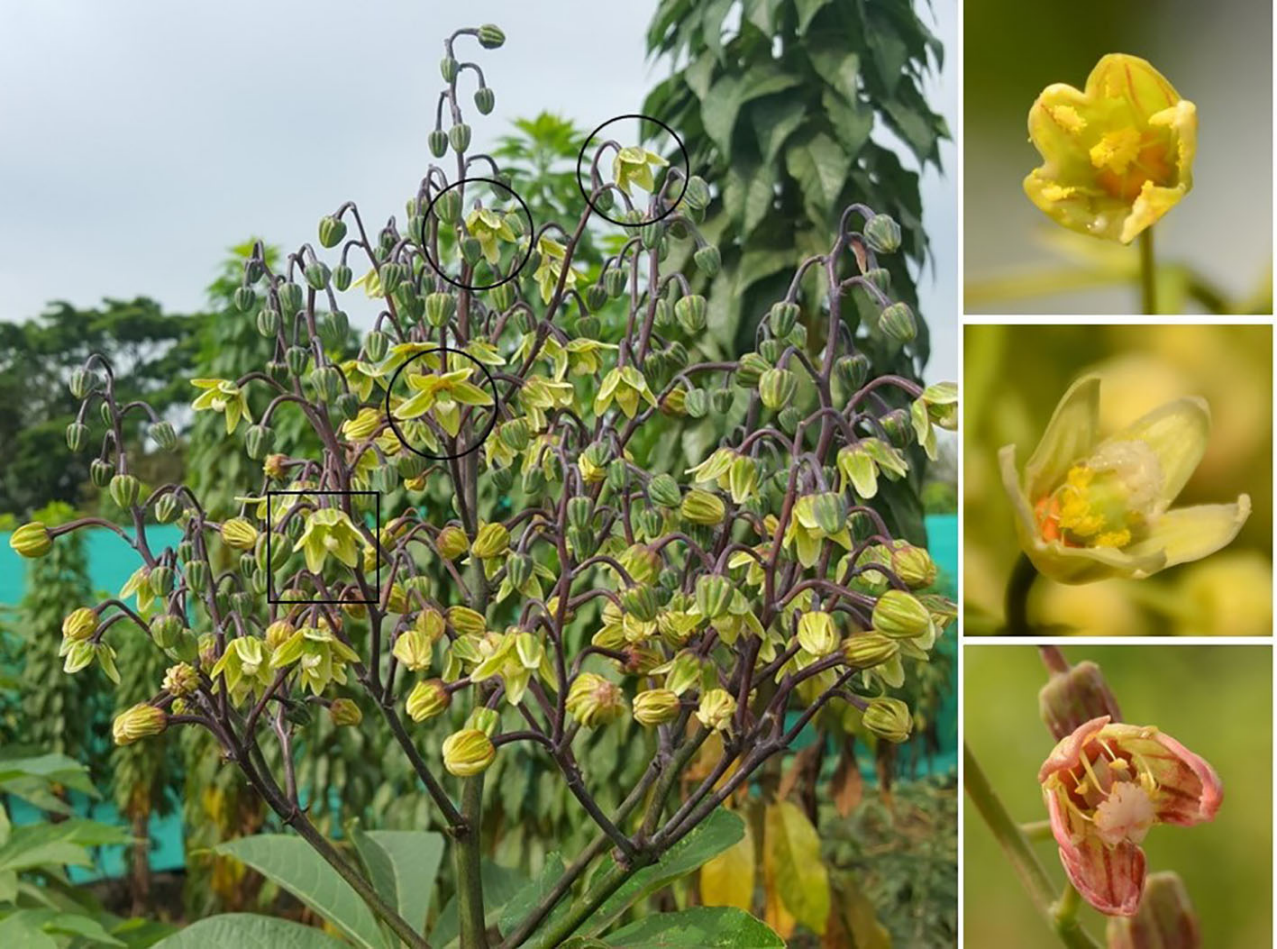
Effect of pruning and application of the plant growth regulator BA. The photograph on the left illustrates inflorescence at the first branching event from SM 3348-29 after pruning and BA application. The flower within the square is hermaphrodite, whereas flowers within circles are female only. The three photographs on the right show a typical male flower (top), a female flower with intermediate (middle), or full development (bottom) of anthers.

Another observed phenomenon arising as a result of pruning was that flowers in the same inflorescence tended to reach anthesis within a few days from each other and with a common disruption of the normal sequence (from bottom to top). This phenomenon is clear in **Figure 7** but is also obvious from **Figure 5**.

Finally, the germination capacity of botanical seed produced after pruning was evaluated in three separated batches following the standard procedures at CIAT. Out of more than 1,200 seeds 85% of it germinated without any abnormal appearance.

## Discussion

It was occasionally observed, within the 10-plant rows, that one or two plants had a very different flowering pattern. For example, nine plants would flower (and branch) three times during the length of the trial but the remaining plant in the plot would not do so, not even once. Similarly, in a given plot all plants except one, would fail to flower. Moreover, this flowering plant may have up to two levels of branching (e.g. flowered twice). These outlaying plants, however, did not prevent identifying clear responses from the different genotypes to the light treatments, but explain some apparent inconsistencies in the data presented. They were included, however, in the respective plot data.

The nested approach to analyze data in **Table 2** allows for a series of dichotomous decisions illustrated in **Figure 3**. The first contrast (DN *vs* EP) clearly indicates that extended photoperiod has a positive effect and results in a significantly higher number of fruits and seeds. The contrast between the performance under DN *vs* EP in each of the four genotypes agreed with earlier reports (Pineda et al., 2018). EP is a pre-requisite for pruning clones that do not flower under normal conditions. A second decision that the researcher must make relates to the convenience of pruning, provided that EP is available and that genotypes respond to it, or else that genotypes would eventually flower under ordinary conditions. For example, under DN, CM 4919-1 flowers for the first time around 9 months after planting in CIAT experimental station. Pruning can be done whenever plants from this genotype flower under DN, but EP would allow pruning much earlier. The earliest experience pruning young branches was on cassava germplasm adapted to the Guangxi province in China. These plants were grown under DN, in a lowland environment but at a higher latitude (which offer a wider variation in the duration of photoperiod through the year than at CIAT station).

There was a positive response to pruning as data from **Table 2** indicates, corroborating the unpublished experiences in China. A key effect of pruning is to prevent abortion of inflorescences, particularly those produced in the first branching event. A similar result was reported by Hyde et al., in 2019 through the use of silver thiosulfate. This anti-ethylene plant growth regulator failed to hasten flower initiation in cassava, but sustained floral development and prevented their abortion (particularly in the first branching event). Having accepted that pruning is advantageous, then we have to choose between pruning at the first or second flowering event. Results suggest that it is generally better to prune at the first branching event. The final element in an ideal protocol for the production of cassava seed relates to the convenience of spraying BA. Results would recommend the use of this plant growth regulator both in plants pruned in the first or second branching event. These are recommendations that can be made based on the results presented in **Table 2**.

The analysis of the response from different genotypes was not the main purpose of this study which focused in the effect of pruning and application of BA treatments across genotypes (**Table 2**). **Figure 6** was included mainly to highlight that genetic differences seem to matter. Responses of the three genotypes, without pruning under DN and EP agree with parallel studies focusing on the effect of EP alone (Pineda et al., 2018). For SM 3348-29 pruning in the first branching level was clearly the best option. In the case of GM 971-2, on the other hand, pruning in the second flowering event makes sense. Finally, CM 4919-1 would accept pruning at either branching event. Application of BA seems to be particularly useful in late flowering genotypes such as CM 4919-1 and SM 3348-29. Unfortunately, there was not replication for the genotype x pruning combinations in this study. Therefore, future studies should confirm that for genotypes that flower relatively early, pruning in the second branching event without BA application may result in higher seed production than other treatment combinations.

In normal conditions, the percentage of female flowers within a cassava inflorescence is lower than 15% (Kawano, 1980; Ogburia and Okele, 2001). Perera et al. reported in 2012 average percentages of female flowers across three genotypes of about 11%. These authors also reported the occurrence of hermaphrodite flowers, although at a very low frequency (0.2%). There was considerable variation in these figures depending on the genotype. An interesting effect of pruning plants was the generalized feminization of flowers. As a result, there was a sharp increase in the number of hermaphrodite flowers (e.g. male flowers becoming hermaphrodite) as illustrated in **Figure 7**. Moreover, in many cases, what should be male became a female-only cyathia. The feminization of flowers is a positive development and explains the unprecedented number of seeds per plant that could be obtained, for example, in SM 3348-29.

The occurrence of hermaphrodite flowers would facilitate self-pollinations and thus a wider utilization of inbreeding for the genetic enhancement of cassava. The advantages from the use of (partially) inbred progenitors in cassava breeding has been listed (Ceballos et al., 2015; Ceballos et al., 2016; Ceballos and Hershey, 2017). However, hermaphrodite flowers present a new problem. Emasculation of anthers would be required to guarantee the production of hybrid seeds in directed crosses. This is an unprecedented procedure in cassava. There are ongoing efforts to foster the feminization process (e.g. reduce the number of hermaphrodite flowers in favor of female-only cyathia) through increased concentration of BA treatments.

Capacity for successful pruning can be quickly acquired after a short training. It was observed that proper pruning at the first branching event is easier than at the second one, because the chronology of events in the first flowering event is slower than in the second. It was easier, therefore, to detect on time the globular shaped shoots that were ready for pruning in the first flowering event. On the other hand, branches in the second flowering event seem to develop much faster and as a result, some of the pruning was done later than desirable (e.g. pruned branches were already relatively large). The botanical seed produced behaved normally when germinated. This is obviously a key requirement for the protocols suggested in this study to be useful.

## Conclusions

The most important finding of this work is that pruning young branches prevents the abortion of the first inflorescence, fostering seed production much earlier than in untreated plants of late flowering genotypes. EP is fundamental for the induction of the first branching event. Therefore, a combination of EP with pruning is a sensible recommendation. Since breeders are interested in the early production of seed, it seems also reasonable to focus on pruning at the first flowering event. The combination of extended photoperiod, pruning, and BA application was particularly advantageous for late flowering genotypes such as CM 4919-1 and SM 3348-29, which otherwise produce very little or no seed at all. Early flowering genotypes such as GM 971-2 seemed to react differently to these treatments. However, breeders seldom need to foster flowering and seed production in early flowering cassava. Future research should validate these preliminary results regarding the reaction of different genotypes. The results reported in this study were conducted in CIAT Experimental Station in Palmira at about 1,000 m.a.s.l. The reaction to pruning may be different in other locations. The advantages of pruning young branches were first detected near Nanning city in China (lower than 500 m.a.s.l but a higher latitude than that of CIAT) and validated also in greenhouse conditions at Cornell University. Pruning has been successfully used at Kasetsart University in Thailand. This would suggest that the advantages of pruning reported in this study are not restricted to Palmira, Colombia, nor to the germplasm used in the present study.

## Data Availability Statement

The datasets generated for this study are available on request to the corresponding author.

## Author Contributions

MP conducted the experiment, took data, performed the pruning, and participated in the analysis of data. BY and YT were involved in the first evaluation of the effect of pruning branches. NM and SS were in charge of the field management and electric system installation. PTH and TLS contributed with the improvement of the protocol through BA application. HC coordinated the work in Colombia, analyzed data, and wrote the manuscript. TLS participated in writing and editing the manuscript.

## Conflict of Interest

The authors declare that the research was conducted in the absence of any commercial or financial relationships that could be construed as a potential conflict of interest.

## References

[B1] Alves A. A. C. (2002). “Cassava botany and physiology,” in Cassava: biology, production and utilization. Eds. Hillocks R. J. Tresh J. M. Bellotti A. C. (Wallingford, United Kingdom: CABI Publishing), 67–89.

[B2] Cadavid-L L. F. (2012). “Soils and Fertilizers for the Cassava Crop,” in Cassava in the third millennium: modern production, processing, use, and marketing systems. Eds. Ospina B. Ceballos H. (Cali, Colombia: Centro Internacional de Agricultura Tropical (CIAT)), 113–137.

[B3] Ceballos H. Hershey C. H. (2017). “Cassava,” in Genetic improvement of Tropical Species. Eds. Campos H. Caligari P. D. S. (Berlin: Springer), pp. 129–pp. 180, ISBNISBN:978-3-319-59817-8

[B4] Ceballos H. Kawuki R. S. Gracen V. E. Yencho G. C. Hershey C. H. (2015). Conventional breeding, marker assisted selection, genomic selection and inbreeding in clonally propagated crops: A case study for cassava. Theoret. Appl. Genet. 9, 1647–1667. 10.1007/s00122-015-2555-4 PMC4540783 26093610

[B5] Ceballos H. Pérez J. C. Orlando J. B. Lenis J. I. Morante N. Calle F. (2016). Cassava breeding II: The value of breeding value. Front. Plant Sci. 7, 1–12. 10.3389/fpls.2016.01227 27621734 PMC5003041

[B6] Ceballos H. Jaramillo J. J. Salazar S. Pineda L. M. Calle F. Setter T. (2017). Induction of flowering in cassava through grafting. J. Plant Breed. Crop Sci. 9, 19–29. 10.5897/JPBCS2016.0617

[B7] Costes E. Crespel L. Denoyes B. Morel P. Demene M.-N. Lauri P.-E. (2014). Bud structure, position and fate generate various branching patterns along shoots of closely related Rosaceae species: a review. Front. Plant Sci. 5 Article 666, 1–11. 10.3389/fpls.2014.00666 PMC4251308 25520729

[B8] de Bruijn G. H. (1977). Influence of day length on the flowering of cassava. Trop. Root Tuber. Crops Newslett. 10, 1–3.

[B9] Dewitte W. Chiappetta A. Azmi A. Witters E. Strnad M. Rembur J. (1999). Dynamics of cytokinins in apical shoot meristems of a day-neutral tobacco during floral transition and flower formation. Plant Physiol. 119, 111–121. 10.1104/pp.119.1.111 9880352 PMC32210

[B10] Dun E. A. Ferguson B. J. Beveridge C. A. (2006). Apical dominance and shoot branching. Divergent opinions or divergent mechanisms? Plant Physiol. 142, 812–819. 17093134 10.1104/pp.106.086868 PMC1630731

[B11] Gonçalves Fukuda W. M. de Oliveira Silva S. Iglesias C. (2002). Cassava breeding. Crop Breed. Appl. Biotech. 2(4), 617–638. 10.12702/1984-7033.v02n04a18

[B12] Hyde P. T. Guan X. Abreu V. Setter T. L. (2019). The anti−ethylene growth regulator silver thiosulfate (STS) increases flower production and longevity in cassava (*Manihot esculenta* Crantz). Plant Growth Regul. 90 (3), 441–453. 10.1007/s10725-019-00542-x 32214568 PMC7081664

[B13] Jennings D. L. (1963). Variation in pollen and ovule fertility in varieties of cassava, and the effect of interspecific crossing on fertility. Euphytica 12, 69–76.

[B14] Kawano K. (1980). “Cassava,” in Hybridization of Crop Plants. Eds. Fehr W. R. Hadley H. H. (Madison, Wisconsin: ASA, CSSA), 225–233.

[B15] Keating B. (1982). Environmental effects on growth and development of cassava (*Manihot esculenta* Crantz) with special reference to photoperiod and temperature. Cassava News 1 (10), 10–12.

[B16] Ogburia M. N. Okele K. (2001). Hybrid seed production in cassava (*Manihot esculenta* Crantz) after natural and artificial pollination in a humid agroecological zone. Acta Agronom. Hungar. 49 (4), 361–367. 10.1556/AAgr.49.2001.4.7

[B17] Perera P. I. P. Quintero M. Dedicova B. Kularatne J. D. J. S. Ordoñez C. A. Ceballos H. (2012). Comparative Morphology, Biology and Histology of Reproductive Development in Three lines of *Manihot esculenta* Crantz (Euphorbiaceae: Crotonoideae). Ann. Bot. Plants 5 (1), 1–18. 10.1093/aobpla/pls046 PMC3551401 23346343

[B18] Pineda L. M. Morante N. Salazar S. Hyde P. Setter T. Ceballos H. (2018). Induction of flowering I: photoperiod extension through a red lights district. IVth GCP21 International Cassava Conference, Cotonou, Benin June 2018

[B19] Prusinkiewicz P. Crawford S. Smith R. S. Ljung K. Bennett T. Ongaro V. (2009). Control of bud activation by an auxin transport switch. PNAS 106, 17431–17436. 10.1073/pnas.0906696106 19805140 PMC2751654

[B20] Ramos Abril L. N. Pineda L. M. Wasek I. Wedzony M. Ceballos H. (2019). Reproductive biology in cassava: stigma receptivity and pollen tube growth. Communicat. Integr. Biol. 12, 96–111. 10.1080/19420889.2019.1631110 PMC6615524 31308874

[B21] SAS (2008). SAS/STAT 9.1 User"s Guide (Cary, NC: SAS Institute Inc).

[B22] Silva Souza L. Parreira Diniz R. Neves R. de J. Cunha Alves A. A. de Oliveira E. J. (2018). Grafting as a strategy to increase flowering of cassava. Sci. Hortic. 240, 544–551. 10.1016/j.scienta.2018.06.070 30349150 PMC6039848

[B23] Stapleton G. (2012). Global starch market outlook and competing starch raw materials for starches by product segment and region. Cassava Starch World 2012. Centre for Management Technology (CMT) Phnom Penh, Cambodia, 22-24 February

[B24] Steel R. G. D. Torrie J. H. (1960). Principles and procedures of Statistics (New York: McGraw-Hill), 78–80.

